# Design, Synthesis, and Antitumor Activities Study of Stapled A4K14-Citropin 1.1 Peptides

**DOI:** 10.3389/fchem.2020.616147

**Published:** 2020-12-10

**Authors:** Nan Wang, Gang Xie, Chao Liu, Wei Cong, Shipeng He, Yinghua Li, Li Fan, Hong-Gang Hu

**Affiliations:** ^1^Institute of Translational Medicine, Shanghai University, Shanghai, China; ^2^Department of Orthopedics, The Second Affiliated Hospital, Shantou University Medical College, Shantou, China; ^3^School of Pharmacy, Second Military Medical University, Shanghai, China

**Keywords:** A4K14-citropin 1.1, all-hydrocarbon stapled peptides, anti-tumor activity, peptidomimetic, animal toxin

## Abstract

A4K14-citropin 1.1 is a structurally optimized derivative derived from amphibians' skin secreta peptide Citropin, which exhibits broad biological activities. However, the application of A4K14-citropin 1.1 as a cancer therapeutic is restricted by its structural flexibility. In this study, a series of all-hydrocarbon stapled peptides derivatives of A4K14-citropin 1.1 were designed and synthesized, and their chemical and biological characteristics were also investigated. Among them, A4K14-citropin 1.1-Sp1 and A4K14-citropin 1.1-Sp4 displayed improved helicity levels, greater protease stability, and increased antitumor activity compared with the original peptide, which establishes them as promising lead compounds for novel cancer therapeutics development. These results revealed the important influence of all-hydrocarbon stapling side chain on the secondary structure, hydrolase stability, and biological activity of A4K14-citropin 1.1.

**Graphical Abstract:**
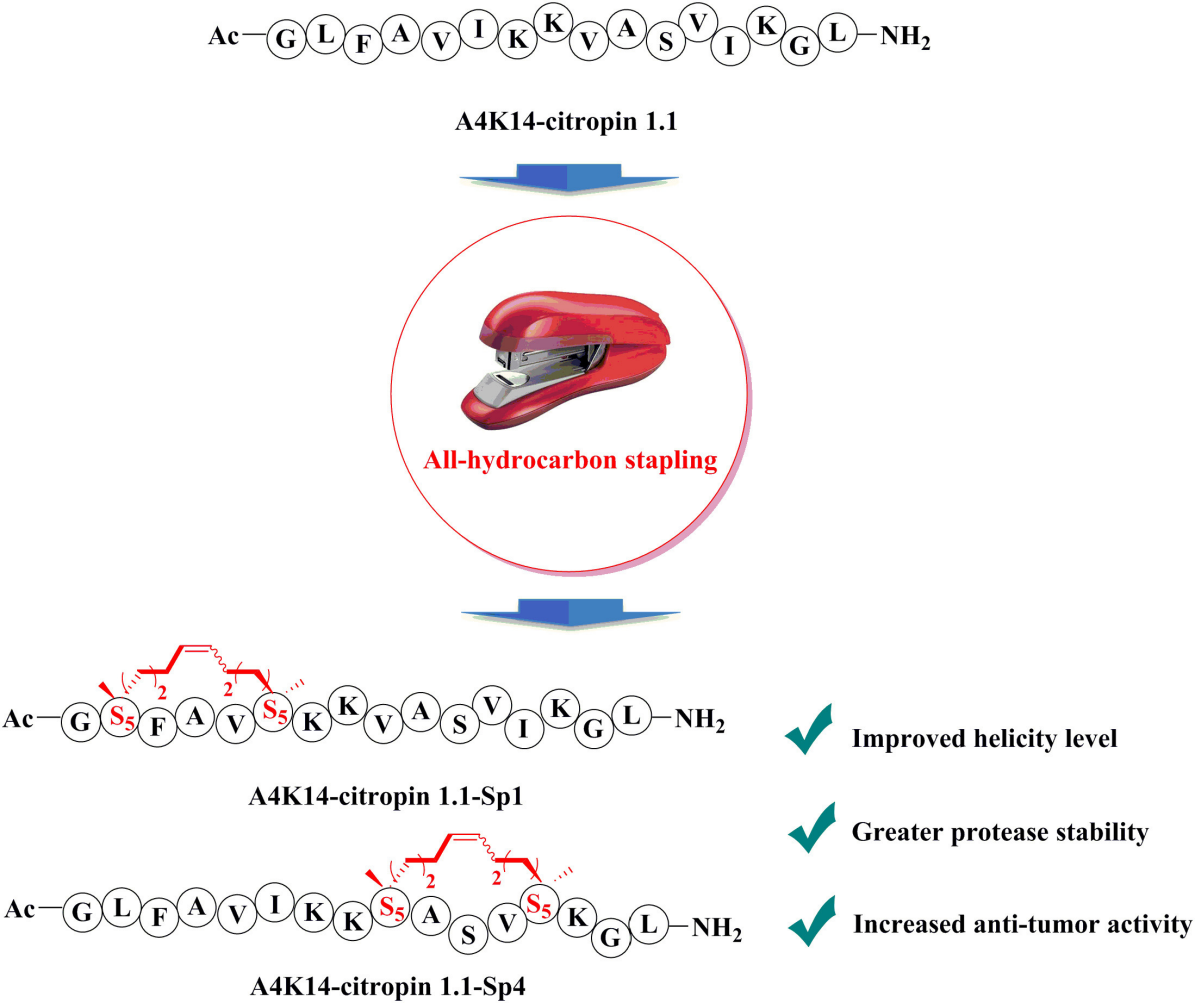


## Introduction

Malignant tumors are one of leading causes of death worldwide investigated by the World Health Organization (WHO) (Murray and Lopez, 2013). There were more than 12 million cancer cases and 7 million cancer deaths occurring in both male and female individuals in 2008 worldwide; the same numbers reached 15 and 8.8 million, respectively, in 2015 (Torre et al., 2011). Cancer has, therefore, become a major public health issue and a leading cause of human mortality (Kobayashi et al., 2002; Ross and Small, 2002; Tong et al., 2018). While small-molecule anticancer drugs have achieved a certain effect, the application of chemotherapy is restricted by a large number of side effects, for example, liver, kidney, and gastrointestinal toxicity; hair loss; diarrhea; breathing troubles; and respiratory difficulties (Lee et al., 2013). Because of their interesting chemical structures and extensive biological activity, peptides have attracted a significant amount of attention (Henninot et al., 2018).

The antimicrobial peptide (AMP) belongs to the membrane-active peptides family that exhibits antibacterial, antitumor, antiviral, and other biological activities due to its cell membrane perforating ability that destroys the structure of the cell membrane and then causes intracellular material leaking out and ultimate cell death (Lohner, 2017). Citropin (GLFDVIKKVASVIGGL), obtained from amphibians' skin secreta, is an α-helix containing 16-residue AMP. A previous study showed that Citropin exhibits broad biological activities such as antibacterial, antitumor, and neuronal nitric oxide synthase (nNOS) inhibition. In a structure activity relationships (SAR) study, Bowie and his team found that replacement of Asp4 and Gly14 with Ala and Lys (termed A4K14-citropin 1.1) resulted in a more stable α-helix than Citropin on the C-terminal section, and it led to better biological activities (Doyle et al., 2003). Hence, A4K14-citropin 1.1 (GLFAVIKKVASVIKGL) has become a potential antitumor leading compound. However, linear peptides hardly retain their native conformation and binding capability owing to their poor structural reinforcement, and they cannot resist proteolytic degradation and cross the cell membrane, which were closely related to AMP's biological activity because of their cell-membrane hole-punching mechanism (Marr et al., 2006; Mourtada et al., 2019). Therefore, a linear peptide itself is a poor therapeutic candidate.

It was reported that increased helicity, hydrophobicity, and penetrating ability of AMP within a certain range could improve their biological activity (Pouny et al., 1992; Dathe et al., 1997; Wieprecht et al., 1997; Dathe and Wieprecht, 1999; Avrahami and Shai, 2002; Shang et al., 2012). For these reasons, we believe that synthetic methods reinforcing their native α-helix conformation, thereby restoring binding affinity, is an effective strategy for AMP optimization. Among these methods, peptide stapling is one of the most established ones for generating α-helices. The macrocyclization process could improve structural rigidity and reinforce the desired α-helical conformation of the peptide, resulting in enhanced protease resistance and improved cell-penetrating ability (Cui et al., 2013; Mortensen et al., 2019; Kannan et al., 2020; Li et al., 2020). Besides, the aliphatic side chain could effectively improve the structural hydrophobicity of peptides. All-hydrocarbon stapled peptides, which involve the ring-closing metathesis of olefin-bearing amino acids developed by Verdine et al., have made the greatest influence on this field so far (Schafmeister et al., 2000). The literature is replete with reports that an all-hydrocarbon stapled strategy is an effective method for peptidomimetics development (Bird et al., 2010; Chapuis et al., 2012; Walensky and Bird, 2014; Liu et al., 2020). In the work reported herein, various derivatives using all-hydrocarbon peptide-stapled strategy were designed and synthesized to increase the cell permeability, membrane aggregation, protease stability, structural hydrophobicity, and antitumor activity of A4K14-citropin 1.1.

## Experimental Section

### General Information

Trifluoracetic acid (TFA), *N,N*-diisopropylethylamine (DIPEA), O-(6-chloro-1-hydrocibenzotrizol-1-yl)-1,1,3,3-tetramethyluronium hexafluorophosphate (HCTU), dichloroethane, first-generation Grubbs' reagent, phenol, triisopropylsilane (TIPS), and diethyl ether were purchased from Acros, TCI, Adamas. All Fmoc-protected amino acids were bought from GL Biochem Shanghai Co. Ltd. Rink amide resin (loading 0.15 mmol/g) was purchased from Tianjin Nankai Hecheng S&T Co., Ltd. Dichloromethane (DCM), dichloroethane (DCE), *N,N*-dimethylformamide (DMF), and acetonitrile used were bought from Sinopharm Chemical Reagent Co. Ltd. Peptides were analyzed and purified by reverse phase high-performance liquid chromatography (HPLC) (RP-HPLC, Shimadzu) using C18 column. The solvent systems were buffer A (0.1% TFA in CH_3_CN) and buffer B (0.1% TFA in water). High-resolution mass spectra (HR-MS) were measured on a Waters Xevo G2 QTOF mass spectrometer. Circular dichroism data were recorded using a JASCO J-820 spectropolarimeter (JASCO Corp., Ltd).

### General Procedures for the Fmoc Solid-Phase Peptide Synthesis

Peptides were synthesized with Fmoc solid-phase peptide synthesis (SPPS) on rink amide resin (initial loading = 0.15 mmol) manually. Fmoc deprotection was realized using 20% piperidine in DMF for 10 min at room temperature. Amino acids (0.45 mmol) coupling was carried out by HCTU (0.45 mmol) and DIPEA (1.35 mmol) in DMF solution for 30 min at room temperature. Olefin metathesis reaction was realized using first generation Grubbs' reagent (0.45 mmol) in dry dichloroethane solution for 4 h at room temperature. Peptides cleavage was carried out by B cocktail (TFA/TIPS/phenol/H_2_O = 88:5:5:2, v/v/v/v) for 2 h at room temperature. Then, the cleavage cocktail was collected, and the chilled diethyl ether was added. The resulting crude peptide was analyzed and purified by RP-HPLC.

**A4K14-Citropin1.1**

169 mg, 67% yield. HR-MS m/z calcd. for C_81_H_142_N_20_O_18_ 1,683.0811; found [M+2H]^2+^ = 842.5535; [M+3H]^3+^ = 562.3758.

**A4K14-Citropin1.1-Sp1**

161 mg, 63% yield. HR-MS m/z calcd. for C_83_H_142_N_20_O_18_ 1,707.0811; found [M+2H]^2+^ = 855.0585; [M+3H]^3+^ = 570.0455.

**A4K14-Citropin1.1-Sp2**

161 mg, 62% yield. HR-MS m/z calcd. for C_85_H_146_N_20_O_18_ 1,735.1124; found [M+2H]^2+^ = 869.0642; [M+3H]^3+^ = 579.7188.

**A4K14-Citropin1.1-Sp3**

186 mg, 71% yield. HR-MS m/z calcd. for C_86_H_148_N_20_O_18_ 1,749.1280; found [M+2H]^2+^ = 876.0834; [M+3H]^3+^ = 584.4036.

**A4K14-Citropin1.1-Sp4**

170 mg, 66% yield. HR-MS m/z calcd. for C_84_H_144_N_20_O_18_ 1,721.0967; found [M+2H]^2+^ = 862.0619; [M+3H]^3+^ = 575.0512.

**A4K14-Citropin1.1-Sp5**

166 mg, 62% yield. HR-MS m/z calcd. for C_90_H_156_N_20_O_17_ 1,789.1957; found [M+2H]^2+^ = 896.1107; [M+3H]^3+^ = 597.7472.

**A4K14-Citropin1.1-Sp6**

166 mg, 63% yield. HR-MS m/z calcd. for C_87_H_150_N_20_O_18_ 1,763.1437; found [M+2H]^2+^ = 883.0813; [M+3H]^3+^ = 589.0584.

**A4K14-Citropin1.1-Sp7**

162 mg, 62% yield. HR-MS m/z calcd. for C_86_H_148_N_20_O_18_ 1,749.1280; found [M+2H]^2+^ = 875.5751; [M+3H]^3+^ = 584.0531.

### Protease Stability Experiment

Fifty microliters peptides in dimethyl sulfoxide (DMSO) (1 mM) and 1,950 μl α-chymotrypsin in phosphate-buffered saline (PBS) (0.5 ng/μl, pH 7.4, containing 2 mM CaCl_2_) were mixed and incubated at 37°C. After 0, 1, 2, 4, 8, and 12 h, the percent residual peptide was monitored by HPLC.

### CD Spectroscopy Study

Peptides in 50% 2,2,2-trifluoroethanol (TFE) aqueous solution (0.1 mg/ml) were recorded at 20°C in a quartz cell of 10 mm path length. Percent helicity was calculated by the follow equation (Wang et al., 2006):

(1) $$\begin{array}{l} \alpha \;=\;\frac{{\left[\theta \right]}_{222}}{{\left[\theta \right]}_{\max}}\;\times \;100\% \end{array}$$

[θ]_222_ is the molar ellipticity of 222 nm; [θ]_max_ = (−44,000 + 250T)(1 – k/n), k = 4, where n is the numbers of amino acids and T = 20°C.

### Cell Culture and Cell Viability Assay

The human prostate cancer cell line C4-2B was kindly provided and authenticated by Dr. Leland Chung (Cedars-Sinai Medical Center, Los Angeles, CA, USA). The human lung tumor cell line A549, the human breast tumor cell line MCF-7, and glioma cell line U87 were obtained from Shanghai Cellular Institute of Chinese Academy of Sciences (Shanghai, China). All cell lines were maintained in Dulbecco's modified Eagle's medium (DMEM) involving 10% fetal bovine serum (FBS) and 1% penicillin–streptomycin (PS) (complete DMEM) and was cultured at 37°C with humidified atmosphere of 5% CO_2_. Cell viability was examined using the cell counting kit (CCK-8) in accordance with manufacturer's protocol. Cells were seeded in 96-well culture plates at a density of 3 ×10^3^ cells/well at 37°C for 24 h. Next day, cells were treated without or with various concentrations (0.39, 0.78, 1.56, 3.125, 6.25, 12.5, 25, and 50 μM) of peptides for 96 h after which 10 μl of CCK-8 reagent was added to each well and incubated for further 2 h at 37°C. The optical density (OD) was then measured at a wavelength of 450 nm on a Cell Imaging Multi-Mode Reader (BioTek, Vermont, USA), and the average OD values for each sample were analyzed using Image-J software (NIH, Bethesda, MD, USA). The half maximal inhibitory concentration (IC_50_) was calculated by GraphPad Prism v7.0 software (San Diego, CA, USA).

### Scratch-Wound Healing Assay

A549 cell line was allowed to grow until a confluent monolayer was observed. After being serum starved overnight, using a sterilized micropipette tip, a linear scratch wound was created across the diameter of the well, splitting the cell monolayer in two. Cellular debris was removed by gentle washing with PBS. The remains were then incubated with fresh low-serum (2% FBS) DMEM without or with 5 μM of A4K14-citropin 1.1 or A4K14-citropin 1.1-Sp4. Immediately, phase contrast images were captured for each scratch wound and used as starting reference point (day 0). Further phase contrast images were captured 1 and 2 days after peptides treatment. The reduction in the scratch-wound area was calculated.

### Transwell Migration Assay

The effect of A4K14-citropin 1.1 upon cancer cells migrating was evaluated using the Transwell permeable support filters (8 μm pore size, 12-well format; Corning Inc., Corning, NY, USA). Briefly, 6 ×10^4^ cells/well of A549 cells were seeded into the upper chamber of the Transwell inserts in low-serum (2% FBS) DMEM with the concentration of 0 or 5 μM of A4K14-citropin 1.1 or A4K14-citropin 1.1-Sp4. Next, the lower chambers were full of complete DMEM and cells incubated at 37°C for 24 h. Finally, cells in the upper chamber inserts were gently washed with PBS twice and gently wiped with cotton swabs to remove nonmigrating cells. Migrated cells were washed briefly, fixed, and subsequently dyed and counted.

## Results and Discussion

### Design of Stapled A4K14-Citropin 1.1 Peptides

**Figure 1:**
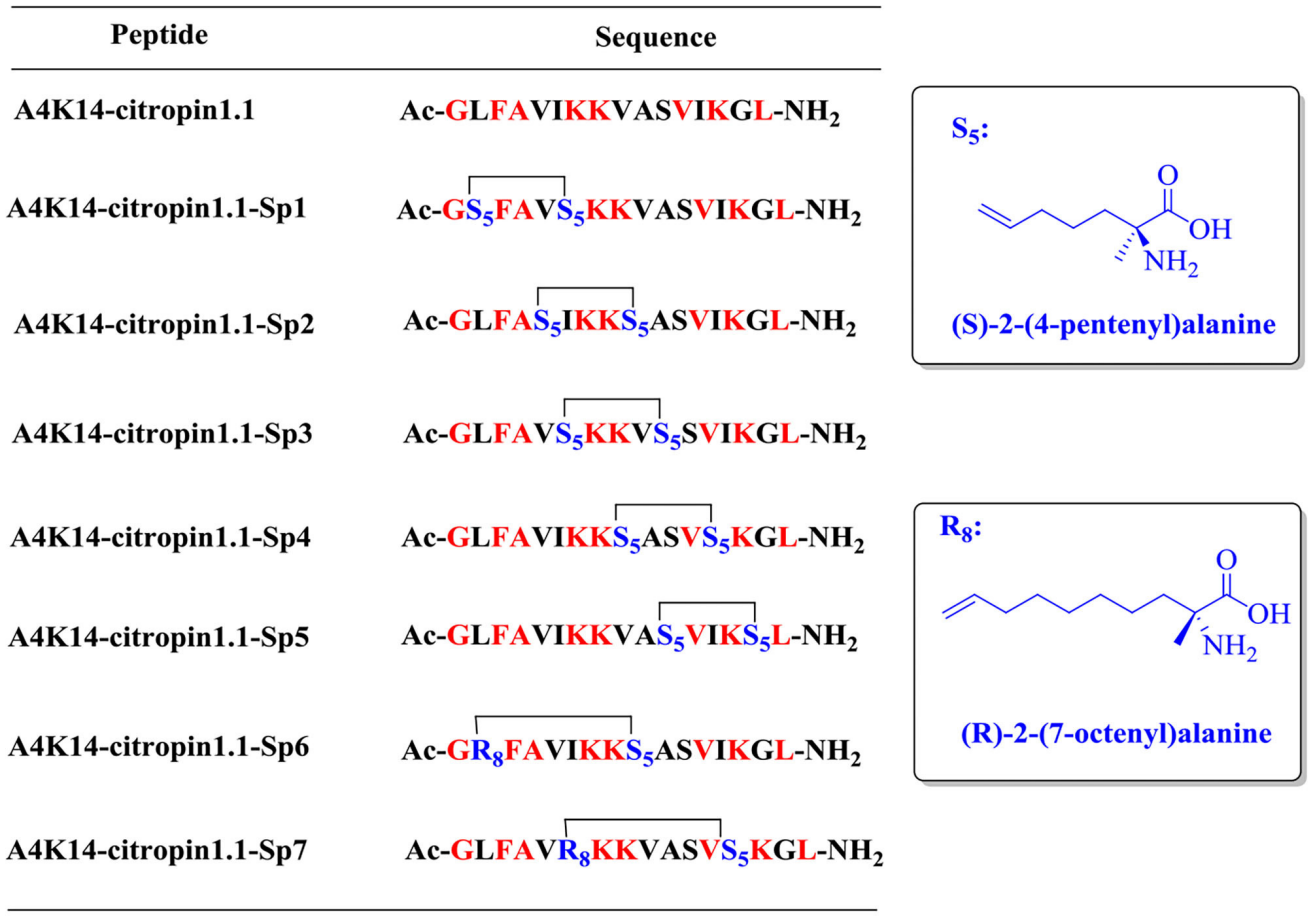
Structures of A4K14-citropin 1.1 and stapled derivatives. The key residues are colored red, and S_5_/S_5_ and R_8_/S_5_ were cross-linked by ring-closing metathesis (RCM).

To design all-hydrocarbon peptidomimetics of A4K14-citropin 1.1, modification of the key residues that are significant for biological activities of A4K14-citropin 1.1 should be avoided. According to a previous study, Gly1, Phe3, Ala4, Lys7, and Leu16 are necessary to remain its biological activities, so in this work, these residues were left intact and A4K14-citropin 1.1-Sp1-5 was designed by introducing (S)-2-(4-pentenyl) alanine amino acid (S_5_) with an i + 4 strategy. Additionally, (R)-2-(7-octenyl) alanine amino acid (R_8_) and (S)-2-(4-pentenyl) alanine amino acid (S_5_) were incorporated at the i and i + 7 positions to obtain A4K14-citropin 1.1-Sp6-7 (Figure 1).

### Synthesis and Characterization of Stapled A4K14-Citropin 1.1 Peptides

**Scheme 1:**
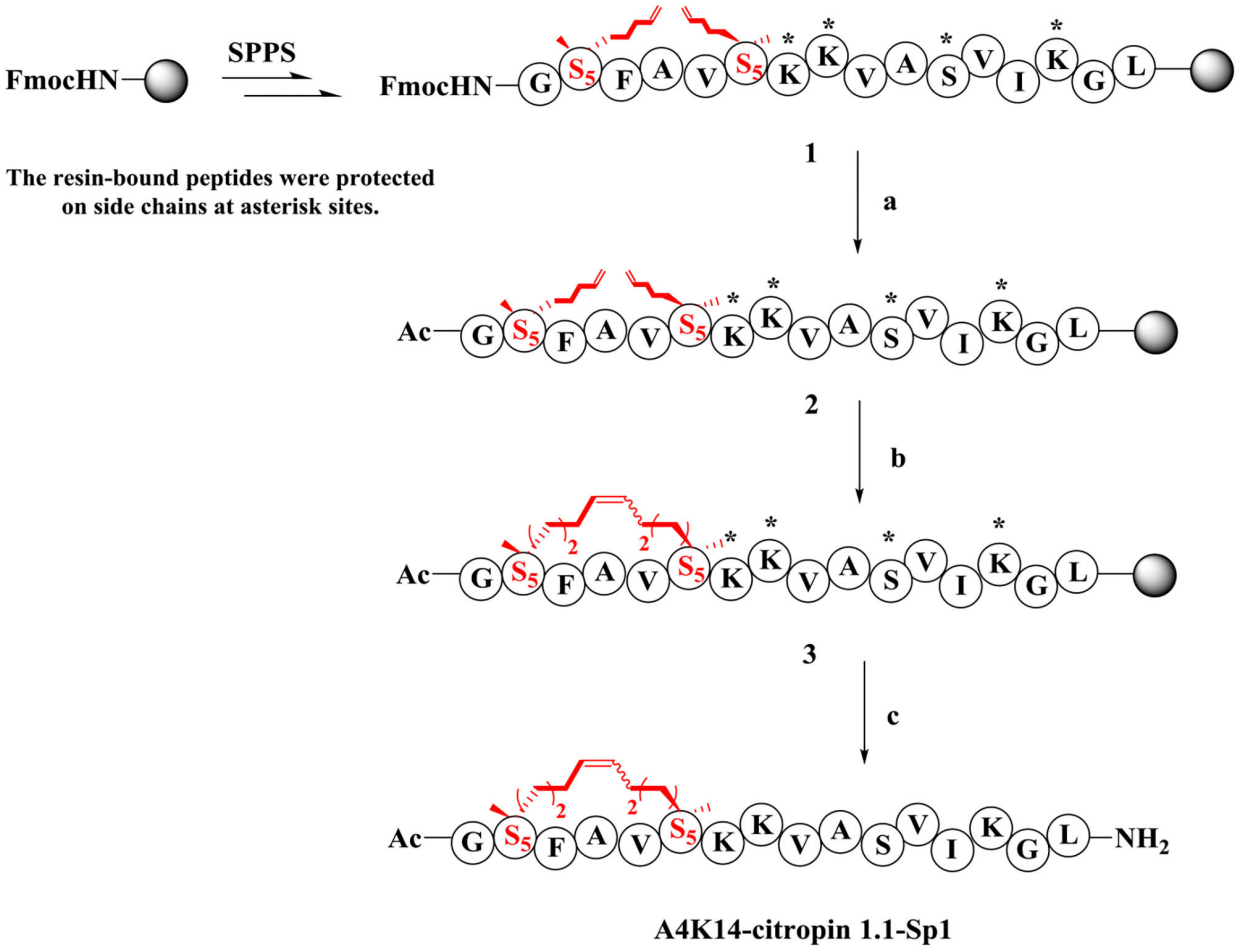
Synthesis route of A4K14-citropin 1.1-Sp1. (a) (i) 20% piperdine/DMF, 20 min, rt; (ii) pyridine/Ac_2_O (1:1, *v/v*), 20 min, rt; (b) first generation Grubbs' reagent, DCE, 2 h, rt; (c) TFA/TIPS/phenol/H_2_O = 88:5:5:2, *v/v/v/v*, 2 h, rt, 63%.

The synthesis of stapled peptides was started from the rink amide AM resin (loading = 0.33 mmol/g) as shown in Scheme 1. Normal amino acids, Fmoc-(S)-2-(4-pentenyl)Ala-OH (Fmoc-S_5_-OH) and Fmoc-(R)-2-(7-octenyl)Ala-OH (Fmoc-R_8_-OH), were introduced into the peptide backbone on resin using HCTU as the coupling reagent to provide Fmoc-protected on-resin peptide 1. After Fmoc deprotection and N-terminal acetylation, intramolecular macrocyclization of on-resin peptide 2 was successfully accomplished with the first-generation Grubbs' reagent in DCE solution to obtain the on-resin stapled peptide 3. Finally, acidic cleavage and concomitant global deprotection using reagent B (TFA/TIPs/phenol/water = 88:5:5:2, *v/v/v/v*) yielded the crude target peptidomimetic A4K14-citropin 1.1-Sp1. Further analysis and purification were achieved by RP-HPLC. Crude products could be easily purified to more than 95% purity, and the yields ranged from 60 to 75%. Then, all of the molecular weights were confirmed by HR-MS and were identical to the theoretical molecular mass.

### Helicity Degree and Protease Stability Analysis of Stapled A4K14-Citropin 1.1 Peptides

**Figure 2:**
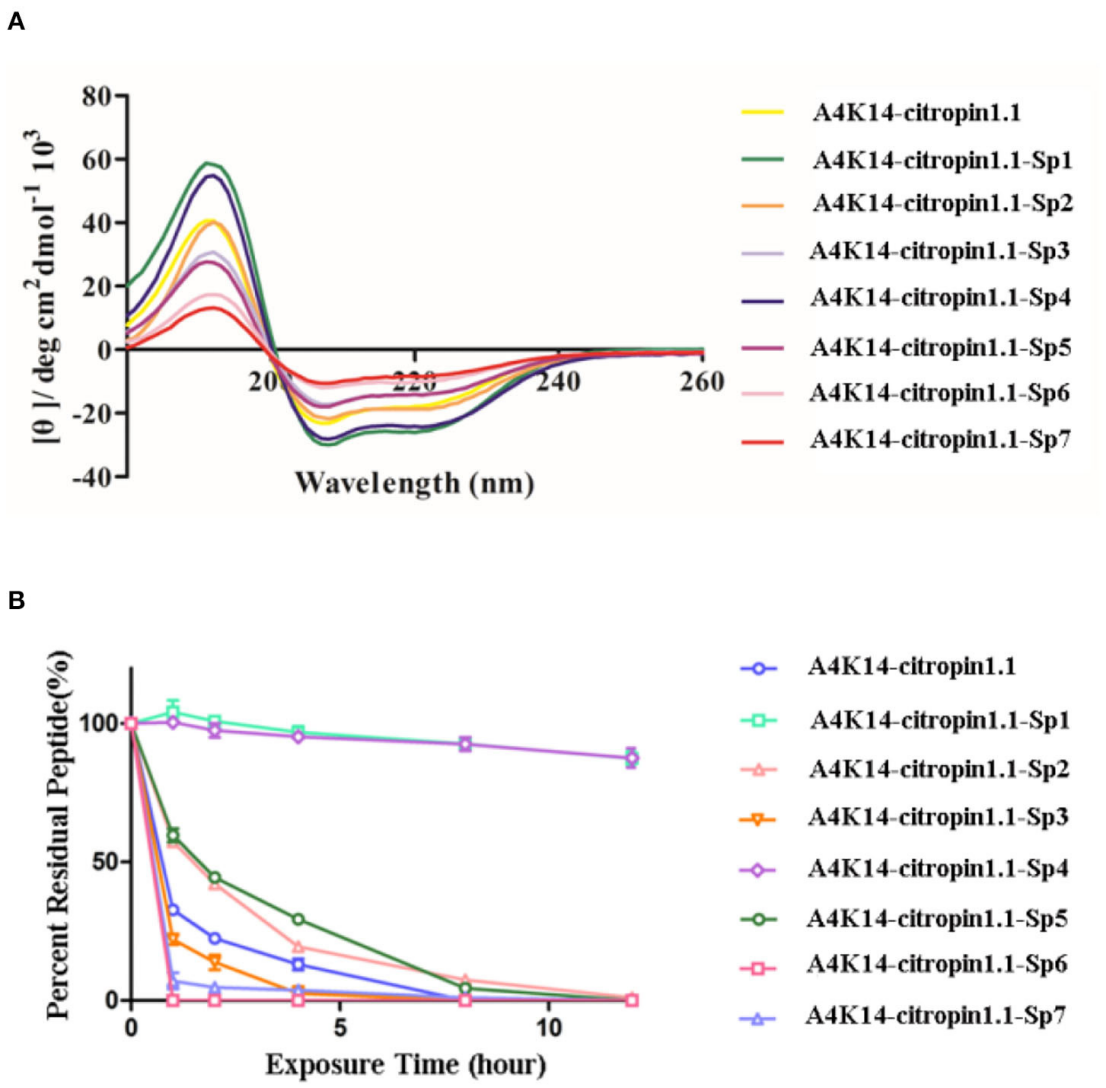
**(A)** The circular dichroism data of A4K14-citropin 1.1 and stapled derivatives. **(B)** Proteolytic stability of A4K14-citropin 1.1 and stapled derivatives under α-chymotrypsin treatment.

The secondary structure of these stapled peptides was measured by circular dichroism (CD). CD analysis indicates that the helicity of initial A4K14-citropin 1.1 was 61.5% and that of the stapled peptides ranged from 13.6 to 89.8% (Figure 2 and Table 1). These results suggested that peptide stapling strategy can effectively optimize the helicity level compared with their linear compartment if the all-hydrocarbon side chains were at the proper position. Among them, A4K14-citropin 1.1-Sp1 and A4K14-citropin 1.1-Sp4 displayed the top 2 degrees of helicity (89.8 and 85.3%, respectively) in the aqueous solution and acquired 1.46- and 1.38-fold improvements compared to A4K14-citropin 1.1, respectively. A further protease stability experiment was conducted using an α-chymotrypsin-mediated degradation test. It was found that, after 12 h of protease exposure, A4K14-citropin 1.1, A4K14-citropin 1.1-Sp2-3, and A4K14-citropin 1.1-Sp5-7 were degraded completely, while more than 85% of A4K14-citropin 1.1-Sp1 and A4K14-citropin 1.1-Sp4 remained intact, suggesting greater protease stability over other peptides (Figure 2 and Table 1).

**Table 1:** α-Helicity, degradation half-life, and antitumor activity of A4K14-citropin 1.1 and stapled derivatives.

**Peptide**	**Helicity (%)**	**t_**1/2**_ (h)^**a**^**	**IC**_****50****_ **(μM)**^****b****^			
			**C4-2B**	**A549**	**U87**	**MCF-7**
A4K14-citropin1.1	61.5	0.63	29.05	14.97	14.8	14.16
A4K14-citropin1.1-1	89.8	>10	8.94	12.48	11.88	11.26
A4K14-citropin1.1-2	66.1	1.72	10.14	12.55	14.76	12.65
A4K14-citropin1.1-3	49.2	0.61	17.89	12.11	11.93	11.92
A4K14-citropin1.1-4	85.3	>10	8.90	10.51	7.277	10.49
A4K14-citropin1.1-5	49.4	1.83	11.90	9.899	8.229	12.42
A4K14-citropin1.1-6	34.9	0.57	35.84	30.19	34.49	23.78
A4K14-citropin1.1-7	13.6	0.58	10.23	16.37	14.72	12.1

a Hydrolysis enzyme degradation half-life.

b *Half maximal inhibitory concentration*.

### Antitumor Activity Test of Stapled A4K14-Citropin 1.1 Peptides

**Figure 3:**
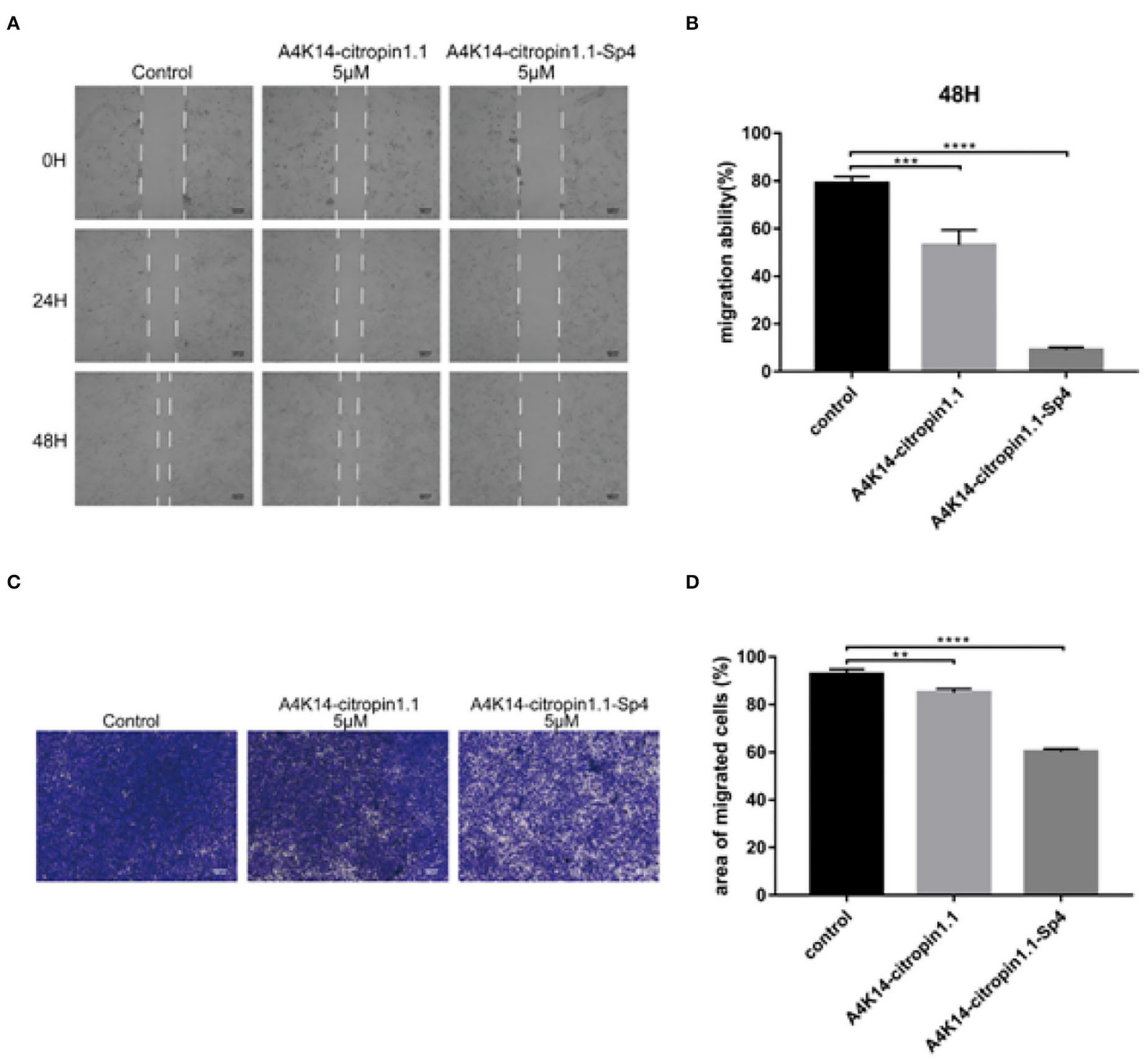
Monolayer A549 cells were wounded using a sterilized micropipette tip subsequently treated with indicated concentration of A4K14-citropin 1.1 or A4K14-citropin 1.1-Sp4 for 48 h; **(A)** the images of wound at each point were recorded. **(B)** The reduction in the scratch-wound area was calculated. **(C)** A549 cells that had passed through the 8-μm polycarbonate membrane without the Matrigel following treatment with the indicated concentrations of A4K14-citropin 1.1 or A4K14-citropin 1.1-Sp4 for 24 h. **(D)** Quantification analysis of the area of the migrated A549 cells. Scale bars: 100 μm (Data are presented as the mean ± standard deviation; *n* = 3; *p < 0.05, **p < 0.01, ***p < 0.001, ****p < 0.0001 vs. the control group).

After A4K14-citropin 1.1 and its analogs were synthesized, their antitumor activities were tested using the CCK-8 test with the human prostate cancer cell line C4-2B, the human NSCLC cell line A549 (adenocarcinoma), the human breast tumor cell line MCF-7, and the glioma cell line U87. The results are summarized in Table 1. It was found that all of the stapled peptides exhibited enhanced activity compared with prototype peptide A4K14-citropin 1.1 (Table 1). Among them, A4K14-citropin 1.1-Sp4 exhibited the best inhibition activity. For further study, we illustrated the inhibitions of A4K14-citropin 1.1-Sp4 upon metastatic characteristic of malignant lung cancer cells by the scratch-wound horizontal-migration (Figures 3A,B) as well as the Transwell vertical-migration assays (Figures 3B–D). In the scratch-wound horizontal-migration assay, A549 cells treated with A4K14-citropin 1.1-Sp4 revealed a better decreased ability to migrate across and close the scratch wound. Similarly, in the Transwell vertical-migration assay, it was found that A4K14-citropin 1.1-Sp4 had a better suppression ability of A549 cells vertical migration than a prototypical peptide at the same dose. Taking CD spectroscopy study and a protease stability experiment into account, it was suggested that protease resistance and stable spatial conformation, which were successfully optimized by stapling strategy, were both significant for the antitumor activity of A4K14-citropin 1.1 and its analogs. Besides, the improved hydrophobicity provided by fatty chains is another possible reason for increased activity.

## Conclusions

In conclusion, a novel series of stapled A4K14-citropin 1.1 derivatives has been successfully realized in a satisfactory yield *via* the standard SPPS strategy and olefin metathesis macrocyclization. *In vitro* data suggested that the analog A4K14-citropin 1.1-Sp1 and A4K14-citropin 1.1-Sp4 displayed improved helicity levels, greater protease stability, and increased antitumor activity compared with the original peptide, which establishes them as promising lead compounds for novel cancer therapeutics development. In addition, considering the close relationship between stable α-helix structure and membrane penetration ability and AMPs' molecular cell-membrane hole-punching role during the killing of pathogens, it was speculated that the antitumor mechanism of A4K14-citropin 1.1 and its analogs is also related to its cell membrane permeation property. Further molecular tracing studies using a fluorescent molecular labeling strategy and relative biological investigation are ongoing, and new findings will be published in due course.

## Data Availability Statement

The original contributions presented in the study are included in the article/Supplementary Materials, further inquiries can be directed to the corresponding author/s.

## Author Contributions

LF and H-GH: conceptualization and design of the study. NW and CL: synthesis of the compounds. GX, WC, and YL: performance of the pharmacological tests. SH: statistical analysis of the data. H-GH: writing and revising of the manuscript. All authors contributed to the article and approved the submitted version.

## Conflict of Interest

The authors declare that the research was conducted in the absence of any commercial or financial relationships that could be construed as a potential conflict of interest.
