# Supplementary material for: Design, Synthesis, and Antitumor Activities Study of Stapled A4K14-Citropin 1.1 Peptides
Source: Front Chem. 2020 Dec 10;8:616147. doi: 10.3389/fchem.2020.616147 (PMC7758422; doi:10.3389/fchem.2020.616147)
Supplement: Supplementary file 1 [file Data_Sheet_1.docx]

Supplementary Material

**HPLC and HRMS spectra of compounds**

**Supplementary Figure 1.** A) The structure of A4K14-citropin1.1; B). The HPLC of purified A4K14-citropin1.1. Gradient: 0-90% of buffer B in 30 min with C18 column (5 *μm*, 2.5 mm×250 mm); C) HR-MS spectrum of A4K14-citropin1.1 calcd. for C_81_H_142_N_20_O_18_ 1683.0811; found [M+2H]^2+^: 842.5535; [M+3H]^3+^: 562.3758.

**Supplementary Figure 2.** A) The structure of A4K14-citropin1.1-Sp1; B). The HPLC of purified A4K14-citropin1.1-Sp1. Gradient: 0-90% of buffer B in 30 min with C18 column (5 μm, 2.5 mm×250 mm); C) HR-MS spectrum of A4K14-citropin1.1-Sp1 calcd. for C_83_H_142_N_20_O_18_ 1707.0811; found [M+2H]^2+^: 855.0585; [M+3H]^3+^: 570.0455.

**Supplementary Figure 3.** A) The structure of A4K14-citropin1.1-Sp2; B). The HPLC of purified A4K14-citropin1.1-Sp2. Gradient: 0-90% of buffer B in 30 min with C18 column (5 μm, 2.5 mm×250 mm); C) HR-MS spectrum of A4K14-citropin1.1-Sp2 calcd. for C_85_H_146_N_20_O_18_ 1735.1124; found [M+2H]^2+^: 869.0642; [M+3H]^3+^: 579.7188.

**Supplementary Figure 4.** A) The structure of A4K14-citropin1.1-Sp3; B). The HPLC of purified A4K14-citropin1.1-Sp3. Gradient: 0-90% of buffer B in 30 min with C18 column (5 μm, 2.5 mm×250 mm); C) HR-MS spectrum of A4K14-citropin1.1-Sp3 calcd. for C_86_H_148_N_20_O_18_ 1749.1280; found [M+2H]^2+^: 876.0834; [M+3H]^3+^: 584.4036.

**Supplementary Figure 5.** A) The structure of A4K14-citropin1.1-Sp4; B). The HPLC of purified A4K14-citropin1.1-Sp4. Gradient: 0-90% of buffer B in 30 min with C18 column (5 μm, 2.5 mm×250 mm); C) HR-MS spectrum of A4K14-citropin1.1-Sp4 calcd. for C_84_H_144_N_20_O_18_ 1721.0967; found [M+2H]^2+^: 862.0619; [M+3H]^3+^: 575.0512.

**Supplementary Figure 6.** A) The structure of A4K14-citropin1.1-Sp5; B). The HPLC of purified A4K14-citropin1.1-Sp5. Gradient: 0-90% of buffer B in 30 min with C18 column (5 μm, 2.5 mm×250 mm); C) HR-MS spectrum of A4K14-citropin1.1-Sp5 calcd. for C_90_H_156_N_20_O_17_ 1789.1957; found [M+2H]^2+^: 896.1107; [M+3H]^3+^: 597.7472.

**Supplementary Figure 7.** A) The structure of A4K14-citropin1.1-Sp6; B). The HPLC of purified A4K14-citropin1.1-Sp6. Gradient: 0-90% of buffer B in 30 min with C18 column (5 μm, 2.5 mm×250 mm); C) HR-MS spectrum of A4K14-citropin1.1-Sp6 calcd. for C_87_H_150_N_20_O_18_ 1763.1437; found [M+2H]^2+^: 882.5806; [M+3H]^3+^: 589.0584.

**Supplementary Figure 8.** A) The structure of A4K14-citropin1.1-Sp7; B). The HPLC of purified A4K14-citropin1.1-Sp7. Gradient: 0-90% of buffer B in 30 min with C18 column (5 μm, 2.5 mm×250 mm); C) HR-MS spectrum of A4K14-citropin1.1-Sp7 calcd. for C_86_H_148_N_20_O_18_ 1749.1280; found [M+2H]^2+^:875.5751; [M+3H]^3+^: 584.0531.
